# Variable Episomal Silencing of a Recombinant Herpesvirus Renders Its Encoded GFP an Unreliable Marker of Infection in Primary Cells

**DOI:** 10.1371/journal.pone.0111502

**Published:** 2014-11-17

**Authors:** Thomas J. Ellison, Dean H. Kedes

**Affiliations:** 1 Myles H. Thaler Center for AIDS and Human Retrovirus Research, Department of Microbiology, Immunology, and Cancer Biology, University of Virginia, Charlottesville, Virginia, United States of America; 2 Department of Internal Medicine, Division of Infectious Diseases and International Health, University of Virginia, Charlottesville, Virginia, United States of America; University of Southern California Keck School of Medicine, United States of America

## Abstract

The availability of reliable recombinant reporter virus systems has been a great boon to the study of Kaposi's sarcoma-associated herpesvirus (KSHV/HHV-8). Unexpectedly, we found that expression of the ostensibly constitutive green fluorescent protein (GFP) marker was progressively lost during unselected passage in primary rat mesenchymal precursor cells (MM), despite efficient maintenance of latent viral gene expression and episomal partitioning. This repression of EF1-α promoter-driven GFP expression appeared to be passage-dependent, however, since functionally immortalized MM cells derived from long serial passage retained stable expression of GFP following rKSHV.219 infection. Chromatin analysis of cultures that we had infected in parallel demonstrated an increase in repressive H3K27 tri-methylation across the viral episome with the exception of the LANA control region in MM cells infected at early rather than late passage post-isolation. The silencing of GFP expression in the MM cells was reversible in a dose-dependent fashion by the histone deacetylase inhibitor valproic acid, further implicating cellular silencing on incoming viral genomes, and underscoring potential differences in viral gene regulation between primary and functionally immortalized cells. Furthermore, using multispectral imaging flow cytometry, we also determined that the extent of GFP expression per cell among those that were positive did not correlate with the number of LANA dots per nucleus nor the extent of overall LANA expression per cell. This suggests a more complex mode of local gene regulation, rather than one that simply reflects the relative intracellular viral copy number. In sum, we have demonstrated the significant potential for false-negative data when using a constitutive marker gene as a sole means of evaluating herpesviral infection, especially in primary cells.

## Introduction

Despite extensive research, the initial susceptible cell type and latent reservoir of natural infection remain unknown for Kaposi's sarcoma-associated herpesvirus (KSHV/HHV-8); the causative agent of the multifocal vascular malignancy Kaposi's sarcoma, as well as several B cell proliferative disorders including multicentric Castleman's disease (MCD) and primary effusion lymphoma (PEL) [1]-[3]. The inefficiency of lytic replication in existing cell culture models has complicated the development of recombinant virus systems for KSHV research, but several laboratories have developed gene-modified isolates that are now in wide use in the field, especially in primary cell experiments. The recombinant reporter virus system most widely employed in the study of KSHV cellular tropism and regulation of the switch to lytic replication has been rKSHV.219 [4], which bears a selection marker, a constitutive green fluorescent protein (GFP) reporter, and a lytic-program specific red fluorescent protein (RFP) expression cassette driven by the robust early lytic PAN promoter. This system has been utilized in many studies of primary human cells, including oral organotypic raft cultures [5], [6], CD34 + cells [7], endothelial cells [8], and tonsilar lymphocytes [9], [10]. While some of these studies extend to xenograft mouse models, other work has also been published in which another recombinant KSHV was used to infect primary progenitor cells derived from rat embryos [11]. More recently, work by Ashlock et al. demonstrated the susceptibility of murine bone marrow to rKSHV.219 infection [12].

Analysis of stably latent episomes in PEL lines such as BCBL-1 and BC-3 have revealed widespread association with histones bearing posttranslational modifications associated with epigenetic silencing, particularly tri-methylation of histone 3 at position 27 (H3K27me3), with the notable exception of regions involved in latent gene expression [13], [14]. Maintenance of this predominant pattern of repression also depends at least in part on the KAP-1/TRIM28 protein, as it is reversible on suppression of its expression [15]. Importantly, the transcriptional control region of the master KSHV lytic switch factor, ORF50/K-Rta, is occupied by histones bearing bivalent modifications, including both the polycomb-group repressive mark H3K27me3 and the activating histone 3 lysine 4 tri-methylation (H3K4me3) modification, a state which renders the episome poised for lytic reactivation [13], [14]. Recent work has started to shed more light on the mechanisms by which the viral genome develops this nuanced pattern of epigenetic regulation from what is initially an epigenetic blank slate, in that KSHV DNA is neither methylated nor associated with histones in the virion [13], [16].

Much of our understanding about KSHV persistence during latent infection is based on studies of KSHV + PEL-derived cell lines, in which the viral genome efficiently replicates and partitions between daughter cells undergoing mitosis. In the context of de novo infection, however, KSHV infection only rarely leads to such a stable interaction with host cells. Indeed, most de novo infection studies have typically shown a predominant pattern of aberrant latency in most cell types examined, characterized by the limited expression of viral genes associated with the viral latent program, along with varying levels of early or immediate-early lytic gene products depending on the cell type infected [17]. Even in the presence of abundant ORF73/LANA expression, the partitioning of viral episomes or plasmids bearing the minimal cis elements for latent replication tends to be quite inefficient [18]. Often, attempts to recapitulate functional latency following de novo infection require drug selection to enforce episome retention, followed by screening for individual cell clones capable of reactivation [19]. Even in these cases, the capacity of these cells for reactivation generally diminishes over time without continued selection. This underscores the limited nature of our understanding of the initial establishment of functional latency in a newly infected cell.

More recently, work from the Gao laboratory showed that primary rat mesenchymal precursor cells (MM cells) were susceptible to KSHV infection, and able to maintain the episome without selection [11]. Positive selection is not necessary for the establishment of this latent state in these primary cells, but the authors reported that it increases its efficiency (personal communication, S.J. Gao). We set out to examine this stable interaction between KSHV and these uniquely permissive primary cells in more detail, in the absence of drug selection. Unexpectedly, we observed a broad, consistent pattern of H3K27-trimethyl-associated epigenetic silencing across the viral genome in these early-passage primary cells, resulting in the widespread loss of eGFP reporter gene expression despite efficient LANA expression and faithful episomal partitioning. By contrast, this silencing phenotype was notably absent from functionally immortalized MM cells after infection. These observations have broad implications for any studies that depend on reporter gene expression as a primary indication of viral infection, since they might vastly underestimate the extent of infection, or overlook critical subpopulations of infected cells in which the viral episome has undergone differential regulation after infection.

## Materials and Methods

### Antibodies and Cell Cultures

MM cells at passage five from isolation were received as a generous gift from S.J. Gao, and maintained in phenol red-free DMEM (Life Technologies) with 10% fetal bovine serum (FBS, Atlanta Biologicals), supplemented with L-glutamine (Life Technologies) and Penicillin-Streptomycin (Life Technologies). MM cultures were generally split 1∶3 every 2-3 days. The iSLK.219 [19] cell line was a gift from D. Ganem (as kindly supplied by A. Wilson), and passaged as above with the addition of puromycin (EMD Millipore, 540222) at 4 µg/mL. HeLa cells (ATCC) were grown in standard DMEM (Life Technologies) with 10% FBS and Pen/strep. For induction experiments, cells were treated with valproic acid (Sigma Life Science, P4543). Monoclonal antibody to KSHV LANA was derived from the LN53 rat hybridoma line, which was a gift from P. Kellam, grown and purified by the Lymphocyte Culture Center at the University of Virginia. Anti-K8.1 antibody was purchased from Advanced Biotechnologies Inc. (13-212); both were directly labeled with the Alexa Fluor 647 Antibody Labeling Kit (Life Technologies, A-20186) as per the manufacturer's protocol. Antibodies for ChIP experiments were as follows: anti-H3K4me3 (Millipore 04-745), anti-H3K27me3 (Millipore 07-449), anti-H3 (Cell Signaling D2B12 XP), and normal rabbit IgG (Santa Cruz, sc-2345).

### Virus production, titer, and infection

Recombinant KSHV was produced by induction of iSLK.219 cultures with 0.2 µg/mL doxycycline hyclate (Sigma D9891) at approximately 80% confluency, with a media change at 48 hours to Opti-MEM (Life Technologies), supplemented with 2% FBS, penicillin-streptomycin and HEPES buffer (Life Technologies) to 20 mM. Five to seven days post-induction, cell supernatants were collected, cleared twice by centrifugation (3000 RPM ×15 min at 4 degrees C), then passed through a 0.45 micron bottle filter (EMD Millipore). Filtered supernatants were then concentrated by ultracentrifugation at 35,000 rpm for 2 hours through a 20% sucrose cushion in 1x TNE (10 mM Tris-Cl pH 7.4, 200 mM NaCl, 1 mM EDTA). Virus pellets were then resuspended overnight in collection media. Titers for concentrated and unconcentrated rKSHV.219 were determined by spinfection of semi-confluent HeLa cells (2500 RPM ×30 min) with viral supernatants at four-fold dilutions in the presence of 8 µg/mL polybrene (Sigma). At the time of spinfection, the cells in replicate wells were counted. After 36-48 hours, infected HeLa cells were harvested, fixed, evaluated for GFP expression by standard flow cytometry, and titers calculated based on percent GFP + for dilutions in the linear range. Infection of MM cells with rKSHV.219 was performed by spinfection as above, but at an MOI of 10-15.

### Flow cytometry

MM cells were harvested by treatment with 0.05% Trypsin-EDTA (Life Technologies), washed with phosphate buffered saline (Life Technologies), then fixed with 2% methanol-free formaldehyde solution (Thermo Scientific, Rockford IL) in PBS for 10 minutes at room temperature (RT). For MIFC analysis, fixed samples were permeablized and washed with PermWash (BD Biosciences), blocked with 4 µg/mL rat IgG (Sigma, I4131) for 20 minutes at RT, then stained with the labeled anti-LANA antibody at a 1∶400 dilution for 90 minutes, then washed 5x with PermWash and counterstained with 0.5 µg/mL DAPI. Standard flow cytometry samples were generally run on a FACSCalibur (Becton Dickinson) with a 10-color upgrade package (Cytek Development), acquired using FlowJo Collectors' Edition (TreeStar). Induction experiments were run on an LSR Fortessa instrument (Becton Dickinson) using Diva 6.0 for collection. Traditional flow cytometry data was analyzed using FlowJo software (TreeStar). Imaging flow cytometry was performed on an ImagestreamX Mark II (Amnis), and collected using INSPIRE. All three instruments were located at the University of Virginia Flow Cytometry Core.

### MIFC analysis

Data from imaging flow cytometry was analyzed with IDEAS v6.0 (Amnis). Acquired cell images were digitally compensated, then gated for focused cells based on brightfield contrast and gradient RMS features, and further on DAPI + single cells by violet (ch7) intensity and aspect ratio. Nuclear masks were derived by morphology mask of the DAPI channel, and combined with the spot mask algorithm applied to the Alexa-647 channel (ch11), using a spot to cell background ratio of 10.0 and a radius of 2. A spot count feature was defined for the nuclear spots and used to quantify LANA dots per nucleus and percentage of MM cells with 1 or more nuclear LANA dots. Total compensated intensity values for GFP (ch2) and LANA (ch11) were exported along with the number of nuclear dots, for each cell bearing at least one nuclear LANA dot for correlation analysis in Microsoft Excel. Exponential, linear, and logarithmic trendlines were calculated in each sample, and the highest R-squared (coefficient of determination) was tabulated.

### Chromatin Immunoprecipitation

Cell cultures for ChIP assay (approx. 10 million cells per sample) were harvested with trypsin-EDTA solution, neutralized by addition of complete media, and fixed for 10 min at RT by the addition of fresh methanol-free formaldehyde to a final concentration of 1%, then quenched by addition of glycine to 125 mM. Fixed cell pellets were stored at -80 degrees Celsius, then resuspended in ChIP Lysis buffer (1% SDS, 10 mM EDTA, 50 mM Tris-Cl pH 8.1), supplemented with Complete protease inhibitor (Roche) and PMSF to 1 mM. Fixed chromatin was sheared over three successive treatments by a Diagenide Bioruptor (30 seconds on/off, 10 cycles, high intensity, 4 deg C), supplemented with Triton X-100 to 1%, cleared by centrifugation (13k rpm/10 min/8 deg C), then diluted with ChIP Dilution Buffer (1% Triton X-100, 2 mM EDTA, 20 mM Tris-Cl pH 8.1, 150 mM NaCl) plus protease inhibitor and PMSF as above before being divided into input and IP fractions. protocol for washes, incubations, elution/purification. Incubation with antibodies (5 µg/sample) was performed overnight at 4 deg C, followed by recovery of complexes for 2 hours at 4 deg C with Protein A/G PLUS-Agarose beads (Santa Cruz Biotechnology sc-2003), which had been pre-incubated with TE buffer (10 mM Tris-Cl pH 8.0, 1 mM EDTA) and sheared salmon sperm DNA at 50 µg/mL (Ambion, AM9680). The beads and complexes were then washed with TSE (0.1% SDS, 1% Triton X-100, 2 mM EDTA, 20 mM Tris-Cl pH 8.1) containing 150 mM NaCl, then again with TSE with 500 mM NaCl, followed by a Lithium Chloride wash (0.25 M LiCl, 1% NP-40, 1% sodium deoxycholate, 1 mM EDTA, 10 mM Tris-Cl pH 8.1). Two additional washes in TE buffer were followed by elution in ChIP elution buffer (1% SDS, 0.1 M NaCH03). Eluted chromatin was supplemented with NaCl to 200 mM, then incubated at 65 deg C overnight to reverse crosslinks. Residual RNA was removed by a 30 minute treatment at 37 deg C with RNAse A, then ChIP DNA was purified using a MinElute PCR Purification Kit (Qiagen, 28004) as per the manufacturer's instructions. ChIP DNA was diluted 20-200x before interrogation by quantitative PCR using the primer sets listed and Power SYBR Green PCR Master Mix (Life Technologies, 4367659) on an ABI Prism 7900 HT instrument at the Biomolecular Research Facility at the University of Virginia. Samples were assayed in quadruplicate or quintuplicate, using a simple Q-test to exclude single outlier wells that fell outside of a 95% confidence interval. The percent of input recovered was determined by relative Ct values of the input fraction and the IP DNA. Specificity of amplification was confirmed by denaturation analysis. Primer pairs were generally selected based on unique target sequence near known promoter regulatory elements when possible, or at the extreme 5' end of the coding sequence in the case of GFP, to avoid inadvertent detection of the cellular EF-1α promoter. Additional specificity controls were also performed using anti-H3 and nonspecific rabbit IgG antibodies as listed above.

## Results

### Early-passage MM cells lose expression of rKSHV.219-encoded GFP over time in culture

**Figure 1:**
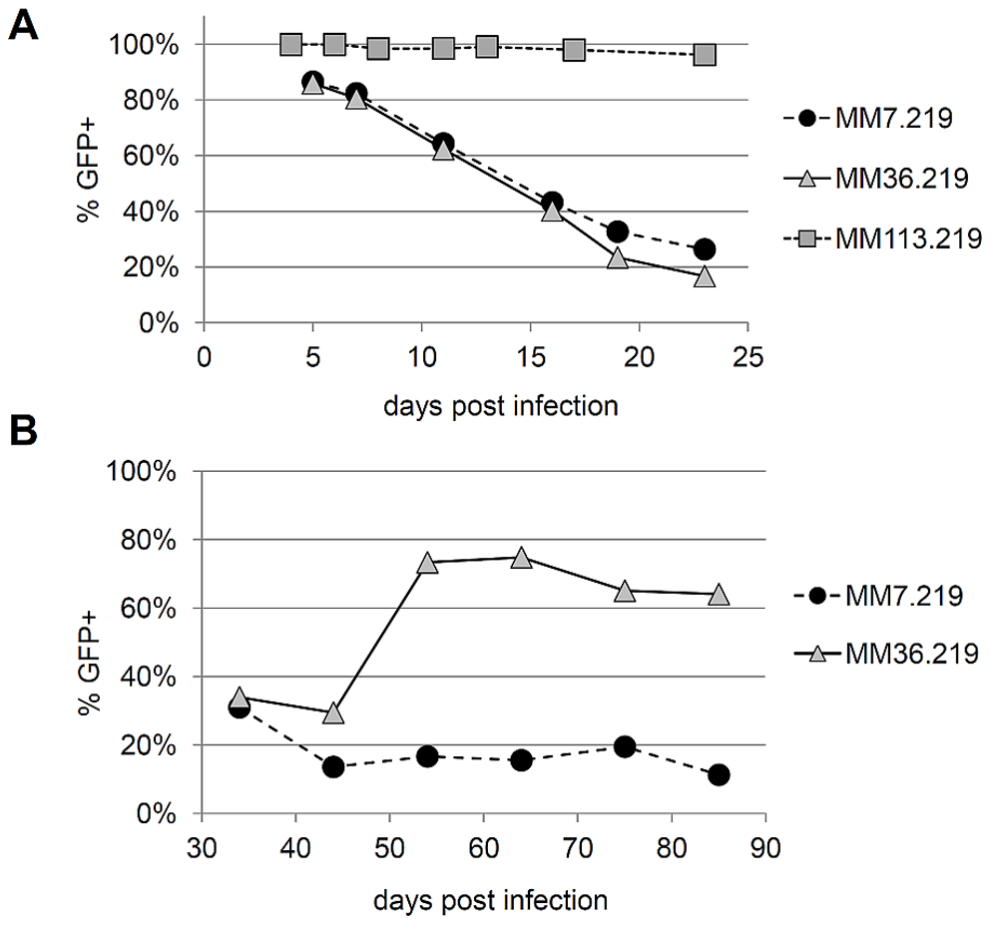
Loss of GFP expression over time in rKSHV.219-infected primary MM cell cultures. Expression of GFP among MM cells infected with rKSHV.219 was evaluated over time post-infection by traditional flow cytometry. (A) The percentage of cells expressing GFP is shown over the first 3 weeks of culture when MM cells were infected at passage 7 (black circles), 36 (gray triangles), or 113 (gray squares). (B) Percentages of MM7.219 (black circles) and MM36.219 (gray triangles) cells expressing GFP after one month of continuous unselected culture.

As Jones et al. previously reported [11], rKSHV.219 efficiently infects MM cells and nearly all cells displayed abundant GFP expression within 24-36 hours of infection following a high multiplicity of infection (MOI 10-15). However, using traditional flow cytometry, we found that in the absence of puromycin selection, the percentage of cells with detectable GFP expression decreased progressively over three weeks of continuous culture (Figure 1A). This loss of GFP signal was evident in MM cells that we had infected early after their isolation (at passage 7 from the embryo) as well as those infected after substantially greater passage in culture (at passage 36 from the embryo). We designated these infected cultures as MM7.219 and MM36.219, respectively. Unexpectedly, under our culture conditions, the uninfected MM cells continued to grow robustly for more than 120 passages, well beyond the previously reported senescence range for these cells (25-30 passages [11]). This enabled us to compare these highly passaged MM cells, which we categorized as functionally immortalized, to the early-passage MM cells. Interestingly, when we infected these MM cells at 113 passages from the embryo (MM113.219), the number of cells retaining GFP expression remained comparatively stable at nearly 100% over four weeks in culture. Of note, there was a decline in the average GFP intensity per cell among of these stably infected MM113.219 cells over passage in culture (∼60% lower MFI at 23 dpi than at 17 dpi), but they remained well above the mock-infected negative control population as interrogated by traditional flow cytometry.

### Higher-passage MM cells spontaneously regain GFP expression

In order to stringently test the stability of KSHV persistence in the MM system, we continued to passage the MM7.219 and MM36.219 cells in the absence of drug selection for more than three months in parallel with uninfected MM cultures. For the MM7.219 cultures, we consistently detected GFP expression in fewer than 34% of the cells during intermittent analyses over nearly 3 months of culture. In contrast, after approximately one month of continuous unselected culture, the majority of the infected MM36.219 cells spontaneously regained GFP expression (greater than 64-75% GFP +, see Figure 1B). This re-emergence of the marker gene was not associated with any significant expression of RFP, as driven by the early viral lytic PAN promoter, suggesting that it was not associated with horizontal spread from spontaneous activation of the canonical viral lytic replication program (data not shown).

### Loss of GFP expression in MM cells does not indicate a loss of KSHV infection

**Figure 2:**
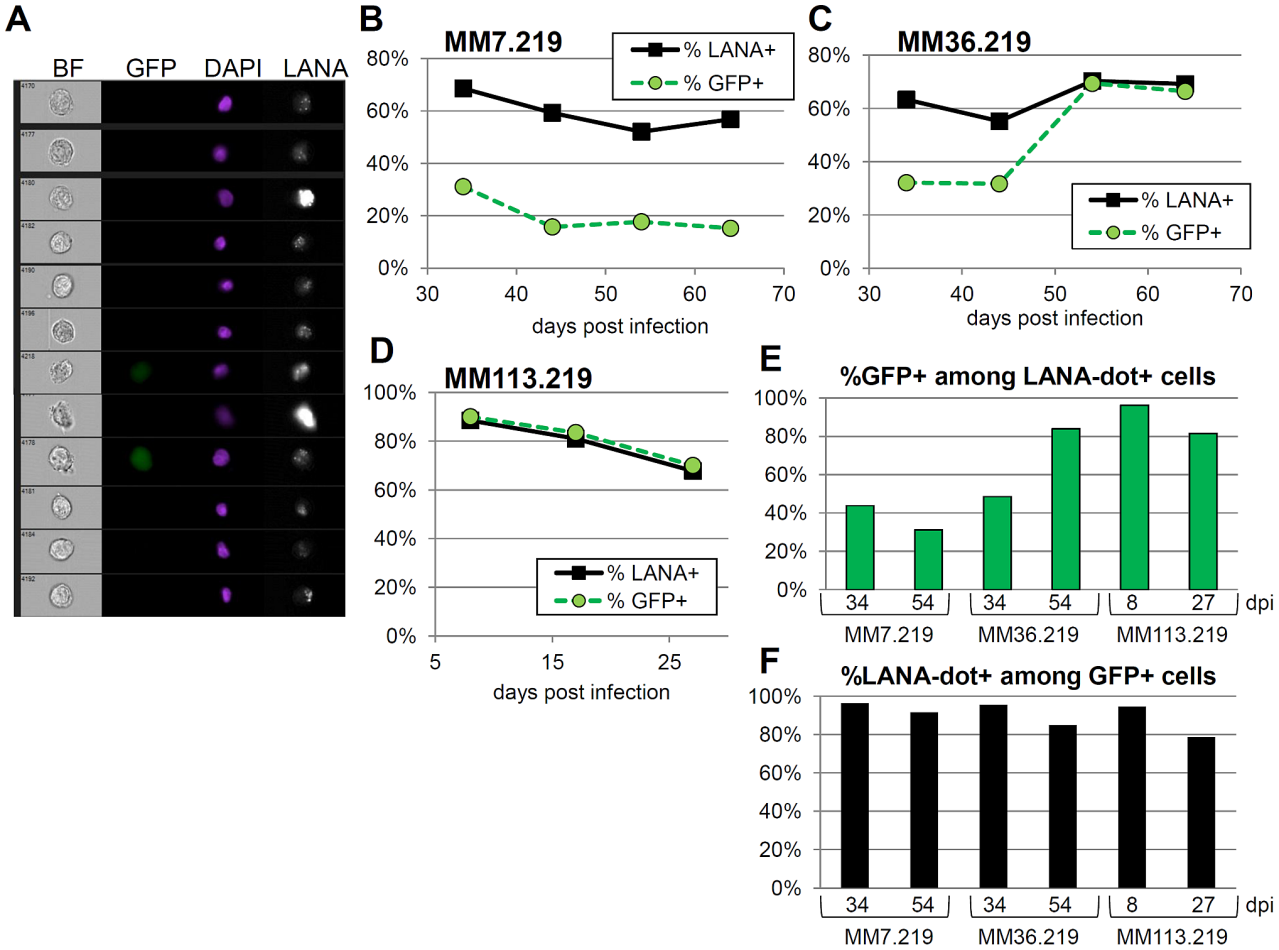
Imaging flow cytometry analysis of LANA expression in rKSHV.219-infected MM cell cultures. Infected MM cells were stained for LANA and assayed by MIFC for the presence of punctate nuclear LANA dots. (A) Representative panel of MM7.219 cells at 21 days post-infection, showing examples of cells by bright-field (BF), GFP, DAPI, and LANA. Percentages of GFP + cells (green circles) are shown over time as compared to the percentage of cells which contain one or more nuclear LANA dots (black squares), for MM7.219 (B), MM36.219 (C), and MM113.219 (D). (E) The specificity of GFP for detecting LANA-dot positive cells is shown across time points and cultures as a percentage of GFP + cells that were also positive for 1 or more nuclear LANA dot. (F) Sensitivity of GFP for detection of infected cells is plotted for the same samples, with percentage of LANA-dot + cells that are also GFP + shown.

Most rapidly growing immortalized cell lines demonstrate inefficient retention and partitioning of KSHV episomes following de novo infection [18]. MM cells, however, reportedly undergo transformation following KSHV infection and efficiently retain multiple copies of the episome even in the absence of positive selection by drug treatment [11]. As a result, we predicted that we would be able to evaluate, at the level of individual cells, the extent of infection in rKSHV.219-infected MM cultures on the basis of characteristic punctate nuclear LANA dots using multispectral imaging flow cytometry (MIFC) [20]-[22]. LANA dots correspond to the presence of the KSHV episome [23], [24], and we have shown previously in HeLa cells that the number of dots per cell correlates with intracellular viral load [20]. As evident in representative images in Figure 2A, abundant LANA staining was present among a majority of the GFP-silent MM7.219 cells, demonstrating efficient latent viral gene expression and retention of episomes even in the absence of the otherwise-constitutive GFP reporter gene expression. Percentages of MM7.219 and MM36.219 cells scoring positive for nuclear LANA dots remained high in both populations over unselected passage in culture, despite dramatic differences in GFP expression (Figure 2B, 2C). Only when GFP expression had reemerged in the MM36.219 cultures at later times post-infection, did this marker begin to correspond with the percentage of LANA-dot positive cells. By comparison, when we infected functionally immortalized MM113 cells at the same MOI, the cultures showed a consistent and tightly corresponding relationship between nuclear LANA dot positivity and GFP expression (Figure 2D). Of note, fewer of the MM113.219 cells scored as GFP + by MIFC than by standard flow cytometry (Figure 1) at later time points due to the lower excitation laser power of the instrument and our use of more stringent threshold gating for GFP positivity in the MIFC assay.

### GFP expression is a variably reliable marker of rKSHV.219 infection

At each time point and condition that we examined, at least a subset of the virus-exposed MM cells was GFP-positive. Among the GFP + cells, the great majority of them (78-96%) also scored as LANA-dot positive by MIFC, confirming the notion that green cells tend to be reliably infected (see Figure 2E). However, the complement was not true: When we examined all LANA-dot + cells for GFP positivity, this concordance varied widely. Beyond the first week of KSHV exposure, GFP served as a poor marker for infection among KSHV-infected MM7.219 cells, with as few as 31% of LANA-dot + cells exhibiting demonstrable GFP expression (Figure 2F). This same discordance between LANA + infected cells and GFP expression was evident for MM36.219 cells for the first month p.i. (Figure 2F). In contrast, infection of functionally immortalized MM cells (MM113.219) resulted in consistent GFP expression among the LANA-dot + cells (81-96%+), similar to MM36.219 cultures beginning after one month p.i. (84%). Importantly, these data demonstrated that GFP was a poor marker for rKSHV.219 in early-passage primary cells, as it may be absent in a majority of LANA + infected cells.

### The extent of GFP expression does not reflect number of nuclear LANA dots nor the overall level of LANA per cell

**Figure 3:**
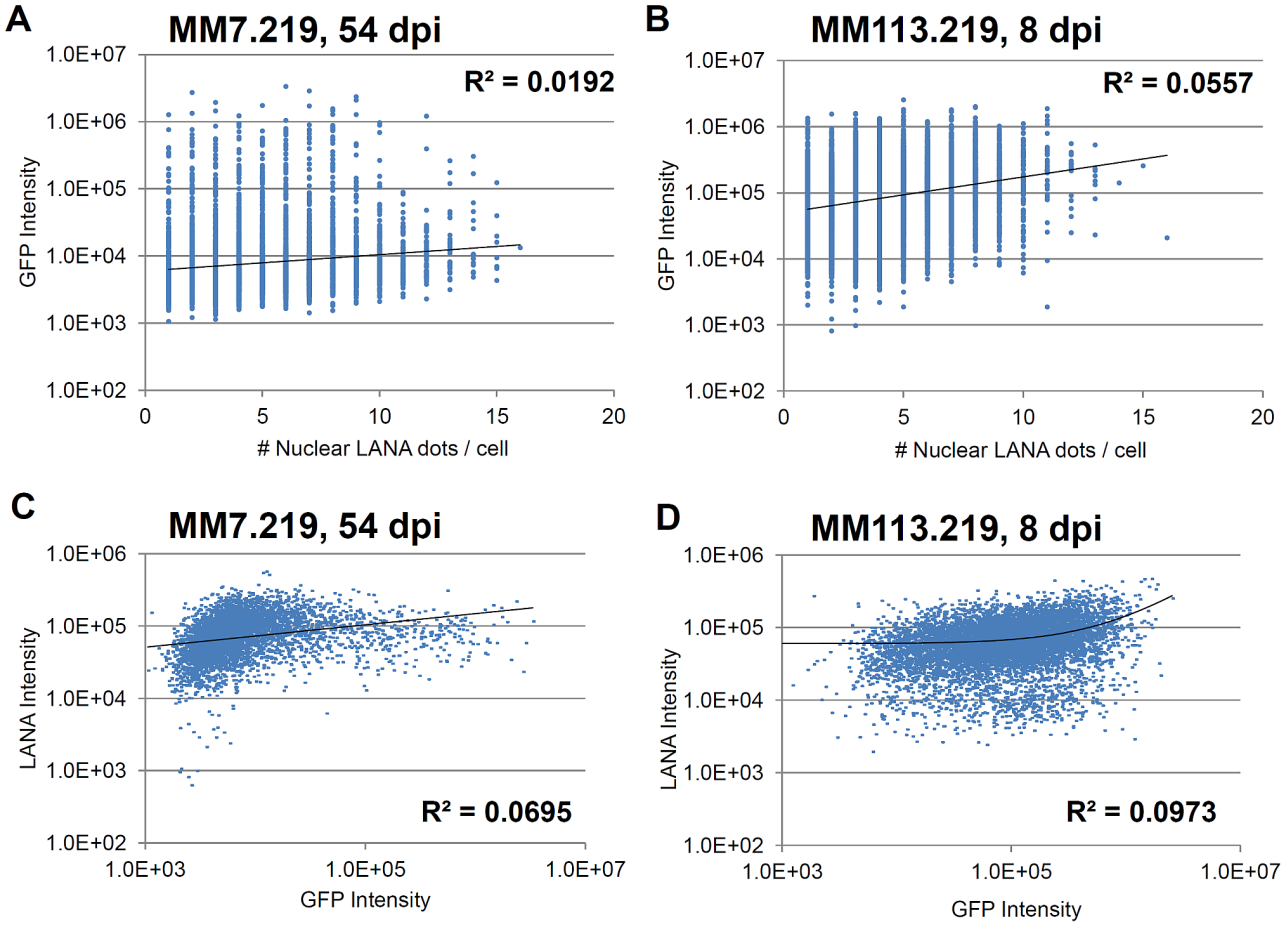
Correlation analysis between GFP intensity, number of LANA dots, and LANA expression. Number of LANA dots per nucleus were exported from MIFC data sets, along with compensated fluorescence intensities for GFP and alexa-647 for LANA detection. For all cells in which one or more nuclear LANA dot was detected, number of LANA dots is shown versus GFP intensity for MM7.219 at 54 dpi (A), and for MM113.219 at 8 dpi (B). Trendlines are displayed in black, and R-squared values shown to represent the correlation between the variables. GFP intensity is also compared to LANA intensity for the same samples (C, D).

To further characterize the discordant relationship between rKSHV.219 infection and GFP expression in MM cells, we compared the intensity of GFP among LANA dot-positive cells to the number of LANA dots per nucleus—a surrogate for episomal copy number [20]. Unexpectedly, the most strongly GFP + cells among the MM7.219 cultures were not those with the highest number of nuclear LANA dots (Figure 3A). Indeed, when we calculated a best-fit trend line for this relationship, there was no correlation (e.g., for MM7.219 at 54 dpi, R-squared value was 0.0192). This independence between degree of GFP expression and LANA dots was broadly evident among infected cells across all time points and conditions tested (no R-squared value higher than 0.1012; see Table 1), even for the MM113.219 cells that showed overall agreement between GFP and LANA-dot positivity (Figure 3B, R-squared  = 0.0557). The lack of correlation between GFP and hallmarks of viral infection also held true when we compared GFP intensity to the degree of LANA stain intensity per cell rather than dot number (Figure 3C and 3D, R-squared values <0.01). This suggested that GFP intensity also failed to accurately predict or represent the overall extent of KSHV latent gene expression per cell. Of note, even if we biased our analyses to favor a correlation by including presumably uninfected cells (no LANA dots) that also showed little to no GFP (rather than just those bearing one or more nuclear LANA dots), the relationship was still weak (maximal R-squared values of 0.2708 and 0.3633 respectively, among the MM36.219 cells at 34 dpi, see the last 2 columns of Table 1). Thus, the number of nuclear LANA dots did not predict whether a given cell would be positive or negative for GFP, suggesting, perhaps, some level of stochastic regulation of the reporter gene locus on a cell to cell basis.

**Table 1:** R-squared values for GFP correlations.

sample	days p.i.	# of LANA dots vs GFP intensity	GFP intensity vs. LANA intensity	All DAPI + # dots vs. GFP intens.	All DAPI + GFP intens. vs LANA intensity
MM7.219	34	0.0294	0.1117	0.1728	0.2685
MM7.219	44	0.0067	0.0022	0.0683	0.1089
MM7.219	54	0.0192	0.0695	0.1482	0.2611
MM7.219	64	0.0152	0.0326	0.095	0.154
MM36.219	34	0.1012	0.2738	0.2708	0.3633
MM36.219	44	0.0199	0.0118	0.0964	0.0239
MM36.219	54	0.037	0.08	0.2029	0.3162
MM36.219	64	0.0318	0.0042	0.1576	0.2121
MM113.219	8	0.0557	0.0973	0.1631	0.2718
MM113.219	17	0.0267	0.0362	0.1279	0.1988
MM113.219	27	0.0375	0.0386	0.1726	0.2293

### Differential epigenetic modifications along the viral episome predict levels of GFP expression following infection of early or late passage primary cells

**Figure 4:**
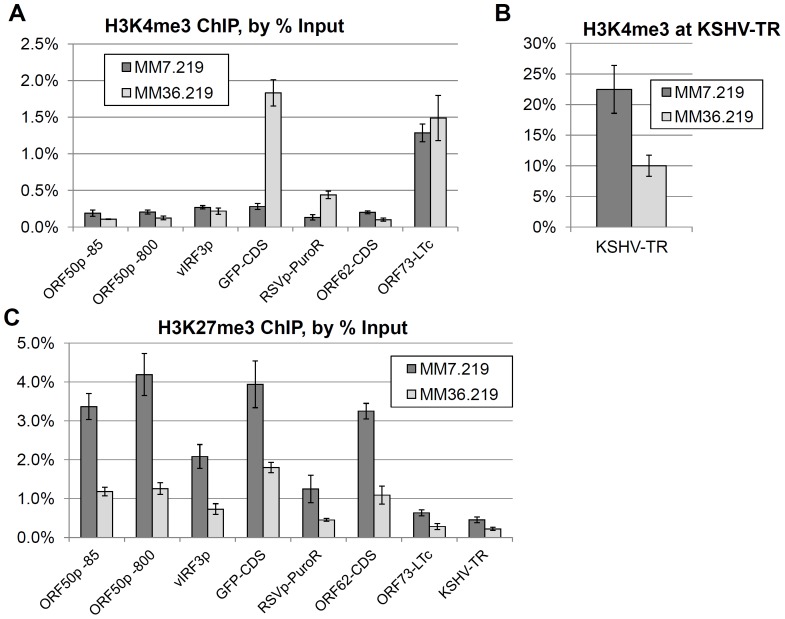
Chromatin Immunoprecipitation comparison of divergent MM7.219 and MM36.219. (A) Enrichment of the activating H3K4me3 histone mark across seven regions of the rKSHV.219 episome at 75 days post infection is plotted as a percentage of ChIP input for MM7.219 (dark gray bars) and MM36.219 (light gray bars). (B) Enrichment of the H3K4me3 mark at the viral terminal repeat for the two cell populations, graphed separately due to scale. (C) Enrichment of the repressive H3K27me3 histone mark across eight positions on the rKSHV.219 episome. Values represent the mean +/- S.D. of 4 to 5 PCR replicates (see Methods).

Based on our observations that the MM7.219 cells broadly retained KSHV infection even in the absence of widespread GFP reporter gene expression, we hypothesized that the recombinant GFP locus might be epigenetically silenced. To investigate this possibility, we subjected the two GFP-divergent rKSHV.219-bearing cultures (MM7.219 and MM36.219) to chromatin immunoprecipitation analysis at 75 days post-infection and examined epigenetic marks at various loci across the viral genomes. We evaluated the enrichment of modified histones at specific regions of the viral episome by quantitative real-time PCR using the primer sets listed in Table 2. As we expected, the major latency promoter that controls LANA expression was enriched for the active-chromatin H3K4me3 epigenetic mark in both cultures, while lytic gene regions such as the ORF50/K-Rta promoter or the late-kinetics ORF62/TRI-1 minor capsid protein gene were not significantly enriched for this mark (see Figure 4A). The GFP reporter gene locus itself showed strong H3K4me3 enrichment in the MM36.219 cells, but not in the MM7.219 cells, consistent with our earlier gene expression results in Figures 1 and 2. Of note, the adjacent puromycin resistance locus was also enriched for H3K4me3 in the MM36.219 culture as compared to the predominantly GFP-negative MM7.219 culture. In the whole-episome tiling ChIP-chip experiments of Gunter et al. [13], the genomic region most highly enriched for H3K4me3 in BCBL-1 and SLKp was the viral terminal repeats (TRs), though the function of this mark at that location, which is presumably not transcriptionally active, remains unclear. Consistent with this report, we also found that the TRs of both cell populations were very highly associated with histones bearing the H3K4me3 mark (Figure 4B). Although this region was the most strongly enriched locus for H3K4me3 in MM36.219 cells, the association was still at less than half the level observed in the MM7.219 cultures. When we instead examined these same viral genomic loci for association with histones bearing the silencing-associated H3K27me3 modification, we observed a broad pattern of association across all regions except the LANA promoter and TRs in the MM7.219 cells (Figure 4C). In contrast, viral episomes among the MM36.219 cultures universally showed a lower extent of enrichment for H3K27me3 at all loci, suggestive of a broad loss of this mode of epigenetic silencing among these cells and consistent with their greater propensity for active expression of GFP.

**Table 2:** Primers used for qPCR analysis of ChIP experiments.

Target	Forward Primer	Reverse Primer	Details	Ref
ORF50p -800	TCCGAGGTAATGTGCTCTATGAAG	ACAGACACCGGAGCAATACCC	Distal ORF50 promoter	[13]
ORF50p -85	TACCGGCGACTCATTAAG	TTGCGGAGTAAGGTTGAC	Proximal ORF50 promoter	[13]
vIRF3p	GCAAGAACATCTGGGGTGAT	AGACAAACATGTGGGCTTCA	KSHV latency gene promoter	
GFP-CDS	CAAGGGCGAGGAGCTGTT	GGACACGCTGAACTTGTGG	5' region of GFP CDS (near EF1α promoter)	
RSVp-PuroR	ATGCCGATTGGTGGAAGTAA	AATGCGGAATTCAGTGGTTC	RSV promoter for Puromycin resist.	
ORF62-CDS	CCTTTATCATGGCCACAACC	TGCCTCGTTGGTTTATCTCC	KSHV TRI-1 capsid protein (late gene) CDS	
ORF73-LTc	AGGGCGGAGTTATATCAAGC	CAGCAACCGGGAGCACAGT	Constitutive latent promoter for ORF73/LANA	
KSHV-TR	GGCGCCCTCTCTCTACTGT	GAGGAGGCTCCCCCAAC	Viral terminal repeat region	

### Silencing of the GFP reporter locus is reversible by treatment with valproic acid

**Figure 5:**
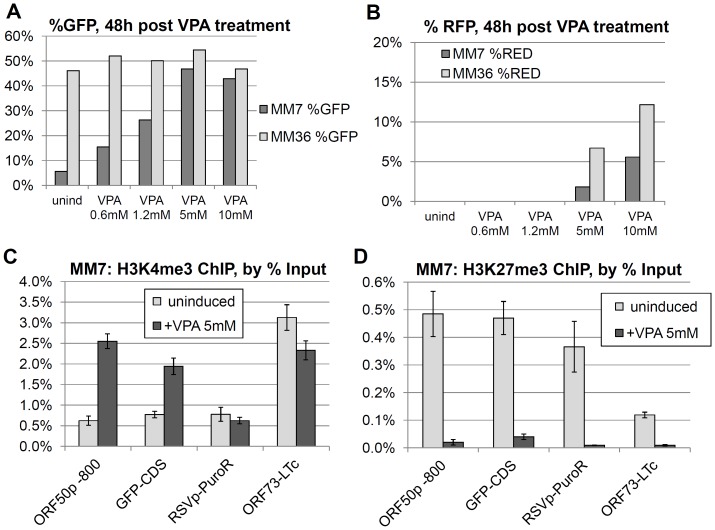
Reversal of MM7.219 GFP silencing by valproic acid treatment. At 79 days post-infection, MM7.219 (dark gray bars) and MM36.219 cultures (light gray bars) were exposed to valproic acid (VPA) at the listed concentrations for 54 hours. The percent cells that were GFP + are shown for the listed doses of VPA (A). The percentage of cells scoring positive for RFP at these doses is also shown (B). In a separate experiment performed 32 days post infection, silenced MM7.219 cultures were treated with 5 mM VPA for 2 days. Enrichment of the active H3K4me3 histone mark across four positions on the rKSHV.219 episome are shown as % of input DNA (C). Similarly, repressive H3K27me3 association is shown for the same four regions (D). Values represent the mean +/- S.D. of 4 to 5 PCR replicates (see Methods).

Reversal of facultative heterochromatin is intrinsic to the reactivation of KSHV from latency, and while the predominant physiological triggers of KSHV reactivation in vivo remain elusive, various drug treatments are capable of inducing the lytic cycle in latently infected cells such as BCBL-1. Recent work has demonstrated valproic acid (VPA) to be among the most potent of these stimulating agents [25]. Although it has pleotropic effects on cells in culture, a primary mechanism of VPA action is through inhibition of a broad variety of histone de-acetylases (HDACs) [26], an activity that rapidly leads to the transcriptional activation of certain silenced genes, by upsetting the balance between activating histone acetylation and repressive de-acetylation. In light of this, we examined the effects of VPA treatment on the MM7.219 and MM36.219 cell populations after their phenotypic (GFP-expression) divergence. Among the MM7.219 cells, treated at day 79 post infection, we observed a dose-dependent increase in the percentage of cells expressing GFP up to 5 mM VPA, with some decrease at higher doses, potentially due to toxicity (Figure 5A). In contrast, the proportion of GFP positive cells remained uniformly high for the MM36.219 cultures subjected to the same VPA dosages. This was consistent with a model in which most of the infected cells in the late MM36.219 culture were already expressing GFP, so the VPA treatment was not able to reverse the phenotype of any GFP-negative infected cells, as was evident in the MM7.219 cultures. Interestingly, the MM36.219 cells did display a dose-dependent increase in the average mean fluorescent intensity per cell following VPA treatment (data not shown). This observation suggested that, even among the GFP + late-passage MM36.219 cells, additional GFP-silenced copies of the viral episome might be present and responsive to the VPA stimulation, contributing to higher levels of average gene expression per cell. Since VPA treatment can initiate the entire lytic reactivation cascade in latently infected PEL cell lines, we also asked whether there was a difference in sensitivity of these two divergent lines to a reactivating agent. To this end, we scored the extent of expression of the secondary reporter gene, RFP among these cells, as a measure of the viral lytic program reactivation under these conditions. We observed appreciable RFP expression only at the two highest doses that we tested (5-10 mM, see Figure 5B). The percentage of cells expressing RFP was uniformly higher among the MM36.219 cells than the more tightly silenced MM7.219 cells. Importantly, neither population showed significant surface expression of the viral late gene K8.1 (less than 0.2%, data not shown) under any of the conditions we tested, suggesting that viral reactivation tends to be abortive or highly inefficient in this cell line, consistent with previous observations [11].

### Reversal of GFP silencing by VPA treatment is associated with loss of repressive H3K27me3 chromatin modifications across the viral episome

To further elucidate the mechanism of the GFP silencing we observed among early-passage MM cells, we focused on MM7.219 cultures 32 days post infection, and repeated the 2-day VPA treatment at 5 mM, this time collecting fixed cells for chromatin immunoprecipitation analysis. We hypothesized that inhibition of histone deacetylase would result in a loss of the repressive H3K27me3 and gain of the active H3K4me3 modifications. As is evident in Figure 3C, VPA treatment resulted in a marked increase in H3K4me3 association at the GFP locus, as well as at the ORF50 promoter. By contrast, neither the RSV promoter driving the puromycin resistance cassette nor the constitutive LANA promoter showed an increase in active H3K4me3 chromatin marks compared to parallel untreated cultures. Dramatically, repressive H3K27me3 histone marks decreased for all loci that we examined (Figure 3D). Furthermore, supernatants from VPA-treated or uninduced cells, as assayed by spinfection of HeLa cells (as we have previously described) showed no evidence of infectious virus (data not shown). Interestingly, the reversal of GFP silencing by VPA treatment appeared to be a transient effect, as we also tested the withdrawl of VPA following the 2-day treatment, and observed that levels of GFP decreased progressively with growth in normal media over 5 days, returning to a level approximately the same as the initial uninduced cultures (data not shown). These results are consistent with a mechanism for VPA action in which it acts broadly to relieve epigenetic repression.

## Discussion

Among actively dividing early-passage MM cells, we observed a marked and progressive loss of GFP expression despite efficient retention of viral genomes and strong viral latent gene expression. Our data suggested that this repression of GFP gene expression likely resulted from an acquisition of a silencing epigenetic phenotype. We found a broad episomal distribution of H3K27me3-modified histones at all sites examined save the LANA promoter and viral terminal repeats, and further, that treatment with an HDAC inhibitor reversed the suppression of GFP. Interestingly, this silencing phenotype appears to be linked to the passage number of the host cells at time of infection since it was not evident following infection of functionally immortalized MM cells. Cumulatively, these findings have broad implications for experimental work that is reliant on consistent marker gene expression, especially in rapidly dividing primary cells.

The initial publication on KSHV infection of this cell type, by Jones et al., demonstrated stable and efficient infection of MM cells in vitro, leading to transformation, as evidenced by tumor formation in nude mice. Our experimental approach differed appreciably from this earlier report in two key aspects. Firstly, we performed our infections and culture of MM cells in the absence of drug selection. Based on our findings, we would expect that the artificial enforcement of an open chromatin state at the selection marker locus through continual drug treatment would preclude development of the GFP-silenced phenotype that we observed since the loci are adjacent in both recombinant virus systems (BAC36 and rKSHV.219). As such, it is of no great surprise that Jones et al. observed a consistently maintained GFP expression among their BAC36-infected cells. Secondly, we did not observe a senescence crisis in our uninfected MM cultures. While our culture conditions varied from theirs to an extent (shallower but more frequent passages), we are unable to easily explain this difference. Our speculation is that our uninfected MM cultures experienced spontaneous functional immortalization over time, gaining, at a minimum, the capacity to significantly outgrow the reported senescence threshold range. The molecular details of this evolution of the MM cell population over time are beyond the scope of this report, However, the observation that uninfected MM cells may, in some cases, substantially outgrow reported senescence complicates the attribution of an immortalizing phenotype to KSHV infection of these cells. We did not, however, test for but doubt we would have found a transformed phenotype for these uninfected and functionally immortalized MM cells.

Recently, Jeffery et al. reported a similar disconnect between GFP and LANA expression in primary human endothelial cells isolated from umbilical cord tissue [8]. In their system, greater proportions of cells expressed LANA than GFP, especially at early time points, and the authors hypothesized that cells receiving a lower number of viral genomes were delayed in their expression of GFP. This work focused on density-limited primary cell cultures within the first ten days of infection and, as such, might be unlikely to undergo the progressive silencing phenotype we observed. Of note, our choice of a high initial MOI (10-15) may well have exceeded the threshold gene dose for reliable early GFP expression as their study hypothesized, though this threshold might also vary by cell type. Regardless, despite this higher MOI, we still observed a loss of GFP over time. More importantly, we found no correlation between the LANA dot number (a surrogate marker for intracellular viral load in other cell types [20]) and the intensity of GFP fluorescence per cell among our continuous cultures of KSHV-infected MM cells. This finding strongly suggests that the relationship between LANA and GFP expression depends on additional factors beyond copy number and time post infection.

An in vivo correlate of the phenotype we observed for KSHV-infected MM cells may be found in the behavior of recombinant murine gamma-herpesviruses at late time points post-infection. Collins and Speck reported that expression of a yellow fluorescent protein reporter (YFP) is routinely lost in vivo among cells infected with a molecular clone of MHV68 following the onset of latency, complicating investigations of the reservoirs of latent infection [27]. The authors further speculated that this shutdown of reporter gene expression is due to epigenetic silencing, and later found it necessary to modify their reporter virus system to include chromatin insulator elements and to express YFP as a fusion protein with histone 2A in order to reliably detect infected B cells in vivo beyond one month post-innoculation [28]. To date, we are unaware of any examples of molecular clones of KSHV with similar refinements to help ensure GFP or other maker expression.

The enrichment of H3K27me3-modified histones across the transgene cassette among the GFP-silenced infected MM cells is strongly reminiscent of the epigenetic pattern present at the corresponding episomal region in naturally infected latent BCBL-1 cells. It is conceivable that this progressive decoration of the lytic gene region of the KSHV episome with reversibly repressive histone modifications is a critical step in the establishment of a functional latent infection, in which cells maintain viral copy number and LANA expression over successive divisions. If so, this might explain, in part, the reported and observed inefficiency of KSHV episomal partitioning and copy number maintenance among typical immortalized cell lines (e.g. HeLa and BJAB), which appear to lack the progressive silencing phenotype we observe in early passage MM cells (Ellison and Kedes, unpublished observation). Regardless, it is unlikely that the acquisition of histone modification patterns resembling those found on latent KSHV episomes in BCBL-1 cells would be itself sufficient to mediate efficient episomal retention, as SLK cells fail to retain KSHV after de novo infection, despite development of canonical histone modifications [13]. Furthermore, although it remains unclear why a small subpopulation of infected early passage MM cells retains GFP expression while the remainder seems to suppress it, it is clear that this phenomenon is independent of the number of nuclear LANA dots (potentially representing the relative intracellular viral load) or overall LANA level.

The primary function of a marker gene such as GFP in a recombinant virus is to provide a reliable indication of the presence of the viral genome within infected cells. Our data clearly demonstrate that the presence of such a constitutive expression cassette in the context of a recombinant herpesvirus genome is not necessarily a guarantee of detectable marker gene expression. Since investigators commonly employ fluorescent protein expression from recombinant reporter viruses to screen for KSHV tropism among both primary cells and established cell lines [29], these observations underscore the need to test more broadly for authentic latent gene expression, even among cells that appear GFP-negative. This potential for false-negative data is an especially major concern among cell types with significant proliferative capacity in vivo, such as activated primary B cell subsets, in which investigators might otherwise overlook authentic latent infection due to marker gene silencing. Critically, the widely used BAC16 molecular clone of KSHV derives from rKSHV.219, and utilizes the same episomal transgene insertion site, promoter, and marker gene [30]. As such, we expect it would have a similar capacity for GFP silencing in some cell types. Although MM cells are likely a poor representation of a physiologically relevant host for KSHV, it is intriguing to observe what appears to be a more nuanced epigenetic regulation of KSHV infection among a primary cell population as compared to closely related immortalized cells. Thus, these findings may help to contextualize the broad disconnect between the behavior of KSHV in established cell lines versus primary cell populations.
